# A CASE OF ASYMPTOMATIC AORTA-ATRIAL FISTULA

**DOI:** 10.51894/001c.123119

**Published:** 2024-08-30

**Authors:** Nicholas Campbell

**Affiliations:** 1 Cardiology McLaren Greater Lansing

## 94

### BACKGROUND

Aorta-atrial fistulas are a rare condition that can be either congenital or acquired. Most of these cases are managed with surgical or percutaneous intervention, leaving only trivial cases to be managed medically.

### CASE DESCRIPTION

A 59-year-old Caucasian asymptomatic male with a history of schizophrenia was referred to cardiology after having a loud systolic murmur auscultated throughout the precordium. A transthoracic echocardiogram was performed and revealed normal left ventricular systolic function, mild left atrial dilation, mild mitral valve regurgitation, a sclerotic trileaflet aortic valve with mild aortic valve regurgitation, dilated ascending aorta measuring 4.8 cm, and moderate to severe tricuspid regurgitation with a right ventricular systolic pressure of 62 mmHg. He was evaluated in the pulmonary hypertension clinic and his murmur was out of proportion to these echocardiogram findings which prompted further evaluation with a transesophageal echocardiogram. This revealed a mobile echodensity beneath the tricuspid valve on the atrial side. A Doppler signal was found and suggestive of a communication between the right atrium and the aorta. The patient underwent a left heart catheterization with an aortogram which confirmed a large fistula between the aortic root and right atrium. A cardiac MRI was also performed to better characterize this.

### DISCUSSION/CONCLUSION

Acquired etiologies of this include vasculitis, trauma, or infection but the fistula was deemed to be congenital. While he has no cardiac symptoms it has been advised to have the fistula surgically closed to reduce longterm left to right shunt and subsequent right heart failure during our Heart Team discussions. This will most likely be closed with a patch along with correction of the ascending aorta and tricuspid valve repair. Aorta-atrial fistulas are rare and have variable presentations making them complex cases to manage. Careful considerations of shunt size and valvular disease must be made to prevent disease progression and additional surgeries.

